# Middle ear pathologies in children living with HIV: A scoping review

**DOI:** 10.4102/sajcd.v69i1.934

**Published:** 2022-11-28

**Authors:** Ben Sebothoma, Minkhenso Maluleke

**Affiliations:** 1 Department of Speech Pathology and Audiology, Faculty of Humanities, University of the Witwatersrand, Johannesburg, South Africa

**Keywords:** children, HIV, middle ear function, middle ear pathologies, scoping review

## Abstract

**Background:**

Middle ear pathologies are associated with and persist in individuals living with human immunodeficiency virus (HIV). Yet, limited research exists on middle ear pathologies in children living with human immunodeficiency viruses.

**Objective:**

To systematically review evidence of middle ear pathologies in children living with HIV, how it is described, measures used to describe it and other relevant information.

**Methods:**

This study was a scoping review. The data were collected from different electronic databases including PubMed, Scopus, Science Direct, ProQuest, and Web of Science. The electronic database search was conducted for articles published between January 2010 and December 2020. Keywords used for searching relevant articles included ‘middle ear pathology’, ‘middle ear disorder’, ‘children’, ‘HIV’, ‘otitis media (OM)’, ‘hearing loss (HL)’, ‘hearing impairment’, ‘paediatric’, ‘minors’, ‘infants’ and ‘HIV/AIDS’. Only articles that were published in English and reported on the middle ear function and pathologies of children living with HIV were considered.

**Results:**

A total of 350 articles were extracted through databases, but only six studies were eligible and included for further analysis. Studies reviewed suggested that middle ear pathologies in children living with HIV exist and are common. Recurrent OM, type B tympanogram, chronic OM and HL with conductive element were common. Tympanometry with a 226 Hz probe tone and air bone gap were used commonly to establish the presence of middle ear pathology.

**Conclusion:**

The findings of this study highlighted that despite the dearth of evidence in this area, available evidence indicates that children living with HIV are at increased risk of middle ear pathology. However, studies in this review have mostly used middle ear measures with poor sensitivity and specificity. Therefore, the prevalence and nature of middle ear pathologies in studies reviewed may have been underreported. Further research using sensitive measures such as wideband acoustic immittance is required. Despite the paucity of evidence, the current findings raise important clinical implications for the assessment and management of middle ear pathologies in children living with HIV.

**Contribution:**

This study makes a significant contribution to the literature regarding middle ear pathologies and HIV, particularly in children.

## Introduction

The human immunodeficiency virus (HIV) continues to be a public health challenge worldwide. In 2018, the prevalence of HIV was estimated to be approximately 37.9 million [32.7-44.0 million] globally, with sub-Saharan countries accounting for the majority of the population living with HIV (Mahy et al., 2019). In the same year, WHO estimated that there were approximately 1.7 million [1.3–2.2 million] children under the age of 15 years living with HIV (Mahy et al., 2019). This is a significant increase from the 2005 data (Joint United Nations Programme on HIV/AIDS [UNAIDS], 2017). Despite the preventative measures that are put in place to curb the spread, the prevalence of HIV in children seems to increase, with evidence of new infections annually.

Despite the efficacy of the antiretroviral therapy (ART) that has been shown to reduce mortality rates of people living with HIV (Gueler et al., 2017; Samji et al., 2013), improve the immune system of people living with HIV, increase life expectancy and improve quality of life (Achappa et al., 2013; Izugbara & Wekesa, 2011; Oguntibeju, 2012), literature suggests that children living with HIV may be at a greater risk of middle ear pathologies (Chao et al., 2012). Evidence suggests that HIV incapacitates the ability of the immune system to fight pathogens and results in opportunistic infections such as middle ear pathologies (Obasineke et al., 2014). Given that children are known to have weaker immune systems and are more prone to ear infections compared with adults (Ramma & Sebothoma, 2016), HIV may have an additional effect on an already weak immune system.

The existence of middle ear pathologies is concerning given that undetected, late detection and untreated middle ear pathologies may cause hearing impairment and other complications. The WHO has stipulated that approximately 50% of individuals with acute otitis media (AOM) will develop chronic suppurative otitis media (CSOM) (Mahy et al., 2019). Further complications of untreated middle ear pathologies include permanent hearing impairment (Hutz et al., 2018), meningitis (Avnstorp et al., 2016), facial paralysis (Lawler, 2018), and vestibular disturbances (Rehagen et al., 2019). The impacts of these conditions in children can have far-reaching consequences.

The impact of hearing impairments in children are well recorded in the literature. Khoza-Shangase and Michal (2014) reported that children whose hearing loss (HL) is identified late are at a greater risk of developmental milestones. Cole and Flexer (2019) reported that children with HL have limited vocabulary, and often struggle to construct a sentence structure. Furthermore, Maluleke et al. (2019) indicated that children with HL have delayed language and inability to communicate, and they struggle to produce clear speech sounds. Some children may experience listening fatigue (Hornsby et al., 2014), especially in classrooms with poor acoustics such as those in low- and middle-income countries (LMICs) (Ramma, 2007), affecting their learning and overall academic achievement (Abraham et al., 2018).

Based on the evidence presented, it is crucial to conduct a scoping review on middle ear pathologies in children living with HIV. Although studies on HIV and other auditory pathologies exist (Dawood et al., 2019), little is known about this current area of investigation, hence the rationale for conducting this scoping review.

## Methods

This scoping review followed a six-step framework proposed by Arksey and O’Malley (2005). The framework includes (1) identifying the research question, (2) identifying relevant publications, (3) study selection, (4) charting the data, (5) collating, summarising and reporting the results. There was agreement on the broad research questions between the two co-authors to be addressed by this scoping review and study protocol together with the identification of keywords and search terms, as well as the selection of databases to be searched. The aim of this study was to integrate the evidence that exists regarding middle ear pathologies in children living with HIV so as to map the literature and acquire an opportunity to find gaps in the evidence and types and sources of evidence to guide practice, policymaking, research and training (Daudt et al., 2013).

### Data sources and search strategy

A search of relevant articles was conducted in the various electronic databases, including PubMed, Scopus Medline, Science Direct, ProQuest and Web of Science. The electronic databases were selected to be comprehensive and to cover the middle ear pathologies in children living with HIV. The search consisted of the following terms: ‘middle ear pathology’, ‘middle ear disorder’, ‘children’, ‘HIV’, ‘HIV/AIDS’, ‘otitis media (OM)’, ‘HL’, ‘hearing impairment’, ‘paediatric’, ‘minors’ and ‘infants’. In line with previous research, the search covered the period from January 2010 to December 2020 (Dawood et al., 2020).

### Inclusion and exclusion criteria

Studies were included in this review if they were written in English, published in peer reviewed journals, and included paediatric population diagnosed with HIV. Furthermore, studies were included if they reported middle ear pathologies in this population.

### Title and abstract relevance screening

During the first level of screening, the title and abstract citations were assessed, while the second level of screening included assessing the abstracts. Lastly, the whole article was reviewed. In that manner, time was not wasted on articles that did not meet the minimum criteria for this scoping review. The titles, abstracts and whole articles were evaluated by both researchers. Duplicates and articles without abstracts were excluded.

### Data characterisation

All the relevant papers or citations for the current scoping review on middle ear pathologies in children living with HIV were reviewed for full publication after the evaluation of titles and abstracts. A spreadsheet was developed by one of the researchers where appropriate publications were confirmed and where the details of publications such as authors, title, year of publication, country, population, methodology, results, conclusion and available data or gaps were recorded.

### Data summary and synthesis

Data were organised in a single spreadsheet and inserted into Microsoft Excel 2016 for descriptive narrative analysis.

### Ethical considerations

This scoping review followed all ethical standards for a study that does not involve direct contact with human or animal participants. However, an ethical waiver (Protocol: STA_2020_23) was obtained from the Human Research Ethics Committee (non-medical) University of the Witwatersrand, Johannesburg, South Africa.

## Results

**FIGURE 1:**
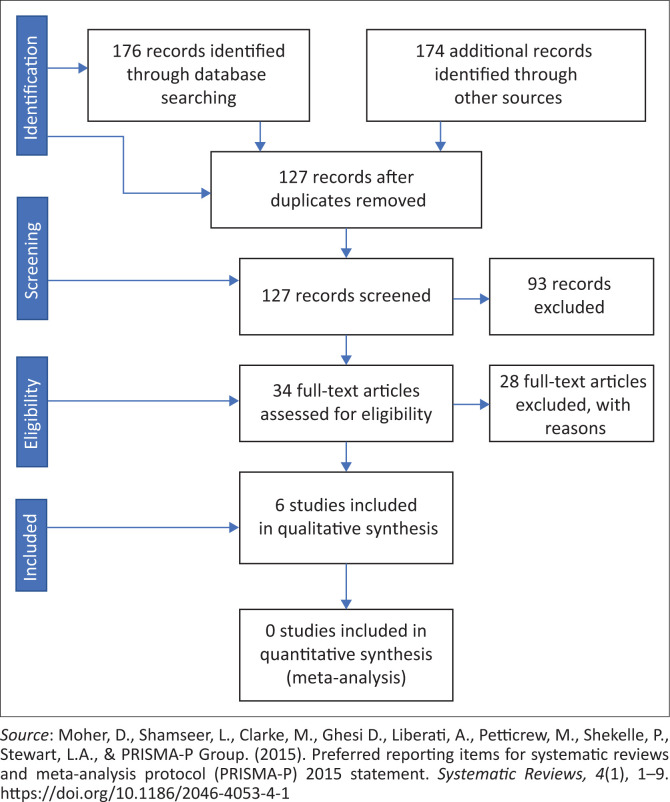
Preferred reporting items for systematic reviews and meta-analysis flow diagram for included.

Figure 1 is a preferred reporting items for systematic reviews and meta-analysis (PRISMA) diagram illustrating the process of the study selection. A total of 350 articles were extracted from different electronic databases. Of these, 127 remained after the removal of the duplicates. The articles were screened based on title and abstract only and 93 were removed at this stage. The 34 remaining articles were included for full-text screening, after which 28 articles were excluded as they did not meet the minimum criteria for this scoping review. The remaining six articles were considered to be relevant to this study and therefore included for further analysis.

### Characteristics of studies

Table 1 summarises the characteristics of the studies reviewed. Three of the studies included in this review employed a case-control design (Maro et al., 2016; Taipale et al., 2011; Torre et al., 2016), while the rest used a cross-sectional design (Hrapcak et al., 2016), longitudinal design (Mwambete & Eulambius, 2018) and a scoping review (Dawood et al., 2020). The total number of samples for all studies combined is 1053 with an age range of 0.8–18 years. All of the studies were conducted in four LMICs countries, particularly in African countries. The researchers included children with HIV-infection and children without HIV-infection and compared the results between the two groups (Maro et al., 2016; Taipale et al., 2011; Torre et al., 2016).

**TABLE 1:** Studies with associated with middle ear pathologies in children living with human immunodeficiency virus.

Authors	Titles	Aims	Methodology	Age	Sample	Context	Results
Torre et al. (2016)	Middle ear function in human immunodeficiency virus (HIV)-infected South African children	To measure the functioning of middle ear using tympanometry in perinatally HIV-infected and HIV-uninfected children living in Cape Town, South Africa.	Case-control	4–14 years	61	South Africa	Parents reported history of middle ear pathologies was higher in children with HIV (34.2%) compared with children who were not HIV positive (25%). Stage IV (WHO classification) HIV positive children are twice at risk of reported middle ear pathologies compared with children with WHO stage II or III classification, however the risk wasn’t statistically significant. CD4 cell count were not associated with a middle ear pathology. Otorrohea was reported in two children with HIV and none was reported in children who were not HIV positive. Six children with HIV presented with type B tympanometry and two of these children had bilateral type B. Two children who were not HIV positive presented with type B tympanometry and one had bilateral type B. All children with type B tympanogram were referred to their physician for further assessment.
Maro et al. (2016)	Auditory impairments in HIV-infected children	To determine the nature of hearing deficits in HIV-positive and HIV-negative children	Case-control	Less than 18 years	244	Tanzania	HIV-positive participants were most likely to have an abnormal tympanogram, history of otorrohea, TB or dizziness. The results were compared between the groups using ANOVA with HIV status and history of ottorohea as a grouping variable. The tests conducted did not differ between the groups, although HIV-positive group had higher number of individuals with hearing level greater than 25dB in the better ear. HIV-positive participants had abnormal DPOAE results at multiple frequencies compared with HIV-negative participants.
Taipale et al. (2011)	Otorhinolaryngological findings and hearing in HIV-positive and HIV-negative children in a developing country	The purpose of this study was to compare otorhinolaryngological (ORL) findings and hearing in HIV-positive and HIV-negative children in Luanda, Angola.	Case-control	Less than 18 years	78	Angola	A total of 92% of ORL pathology was found in children who were HIV-positive and 78% in control children (HIV-negative). The findings in children who were HIV-positive were associated with the following medical conditions: dental caries, cervical lymphadenpathy, facial skin lesions, chronic suppurative otitis media, dry tympanic membrane perforation, tonsils and bilateral hearing loss (greater than 25dB). The ORL pathology in children who were HIV-positive was also associated with otalgia (27%), otorrohea (17%0, TB (2.6%) and pneumonia (2.6%).
Hrapcak et al. (2016)	Hearing loss in HIV-infected children in Lilongwe, Malawi	The purpose of this study was to determine the prevalence and type of hearing loss in HIV-positive children in Lilongwe, Malawi.	Cross-sectional survey	4–14 years	380	Malawi	Their findings indicated that 24% had a hearing loss. Out of that percentile, 82% was a conductive loss, and 4% was a mixed loss. Hearing aid fitting was recommended for children with hearing loss (23%). The prevalence of hearing loss was greater in children with a history of ear infection and otorrohea. Children with a hearing loss were less likely to attend school and had poor emotional and school functioning.
Dawood et al. (2020)	Nature and extent of hearing loss in HIV-infected children: A scoping review	The purpose of this study was to summarise the available peer-reviewed literature on hearing loss in HIV-positive children, specifically to explain the extent and nature of it.	Scoping review	0.3–20 years	17 articles	South Africa	The prevalence of hearing loss differ across the articles (ranges from 6% to 84%). Conductive hearing loss was the most frequent type of hearing loss than sensorineural or mixed hearing loss. Ear infections and ART use was reported as significant in the three of the five articles that reported on significant associates of HIV-related hearing loss.
Mwambete and Eulambius (2018)	High prevalence of antibiotic-resistant otitis media-associated bacterial flora of asymptomatic people living with HIV at Morogoro Hospital, Tanzania	The purpose of this study was to estimate the prevalence of otitis media associated with bacterial flora of asymptomatic people living with HIV on ART and examine the resistance profile of the bacteria.	Longitudinal design	0.2–17 years	290	Tanzania	Ten different species of bacteria were isolated and identified, in which 93.65% of participants living with HIV presented with bacteria that is related to middle ear pathologies, while 6.4% had no ear infections. A total of 3.1% of children had middle ear pathologies and 0.3% of children showed no signs of ear infections. The prevalence rate of bacteria associated with otitis media was 93.4% and a lot of bacteria were resistant to antibiotics. Linear association between the prevalence of OAB with both duration of ART and CD4 counts were observed.

*Source:* Dawood, G., Klop, D., Olivier, E., Elliott, H., Pillay, M., & Grimmer, K. (2020). Nature and extent of hearing loss in HIV-infected children: A scoping review. *International Journal of Pediatric Otorhinolaryngology, 134*, 110036. https://doi.org/10.1016/j.ijporl.2020.110036

Note: Publications that did not meet the minimum criteria for this scoping review were excluded at this phase.

ART, antiretroviral therapy.

### Middle ear assessment methods used

Torre et al. (2016), Maro et al. (2016), Taipale et al. (2011) and Hrapcak et al. (2016) used tympanometry with a 226 Hz probe tone to determine the presence or absence of middle ear pathologies. Ear canal volume (ECV), static compliance and tympanic peak pressure (TPP) were recorded to determine the types of tympanograms. Mwambete and Eulambius (2018) collected ear secretion specimens to identify the bacteria that cause middle ear pathologies. Maro et al. (2016), Taipale et al. (2011) and Hrapcak et al. (2016) included pure tone audiometry (air and bone conduction) using an air or bone gap of 10 dBHL as an indicator to determine the type of HL. A scoping review by Dawood et al. (2020) reported on different types of audiological testing measures including tympanometry and pure tone audiometry. An otoscopic examination was used by Torre et al. (2016), Taipale et al. (2011) and Hrapcak et al. (2016) to rule out the presence of otorrohea because CSOM is characterised by drainage from middle ear (Prabhu et al., 2018).

### Middle ear pathologies

Torre et al. (2016) indicated that out of 37 children living with HIV, 6 (16.21%) presented with bilateral flat tympanograms (Type B), while only 2 children without HIV (8.33%) presented with Type B tympanograms. Maro et al. (2016) also reported significantly high numbers of children living with HIV with abnormal tympanograms. About 25% of children with HIV presented with abnormal tympanograms, while only 12% of children without HIV had abnormal tympanograms.

The prevalence of CSOM in children living with HIV was higher in 0–2-year-old children by 36% (Taipale et al., 2011). Studies by Hrapcak et al. (2016) and Dawood et al. (2020) show a high prevalence of conductive hearing loss (CHL) in children with HIV. According to the study by Hrapcak et al. (2016), 82% of children presented with CHL and 4% presented with mixed hearing loss (MHL). Dawood et al. (2020) reviewed 17 studies, 11 of which reported a high prevalence of CHL. One study reported on the bacteria that causes middle ear pathologies (Mwambete & Eulambius, 2018) and 93.65% of participants with HIV were found to have bacterial flora, which is associated with middle ear pathologies.

Of the six studies reviewed, only three reported on peripheral HL (Maro et al., 2016; Taipale et al., 2011; Torre et al., 2016). Only two studies reported on CHL (Hrapcak et al., 2016; Mwambete & Eulambius, 2018) and one on conductive and MHL (Hrapcak et al., 2016). Out of these six articles, one study reported that CHL is the second most common type of HL in children living with HIV (Dawood et al., 2020). The degree of HL was reported by two studies (Hrapcak et al., 2016; Taipale et al., 2011). Hrapcak et al. (2016) reported that mild, moderate, severe and profound HL are common, while Taipale et al. (2011) only reported on moderate HL. All the articles reported on the HIV factors associated with HL. Ear infection and low CD4 count were the most common factors for middle ear pathologies in children living with HIV.

## Discussion

The purpose of this review was to determine evidence that exists regarding middle ear pathologies in children living with HIV. According to the researchers’ knowledge, this is the first scoping review study that focuses on this particular area and the findings of this study revealed a clear paucity of evidence in this area. Only six studies were relevant for this review, with one study being a review. This paucity of research in this area is concerning given the high prevalence of HIV in children (UNAIDS, 2020) and the association between HIV and middle ear pathologies that have been established in the literature (Khoza-Shangase & Anastasiou, 2020; Sebothoma & Khoza-Shangase, 2020; Tshifularo et al., 2013). The available research indicated that children living with HIV are at an increased risk of middle ear pathologies (Maro et al., 2016; Taipale et al., 2011; Torre et al., 2016) and the prevalence of middle ear pathologies can be as high as 93.65% (Mwambete & Eulambius, 2018). The difference in these studies with regard to prevalence rates may be because of different sample sizes and the assessment methods used, among other factors. Therefore, there is a need for further studies with large sample sizes to determine the prevalence of middle ear pathologies in children living with HIV.

Furthermore, while the studies reviewed indicated that the prevalence of middle ear pathologies is potentially high, all these studies used tympanometry with single probe tones such as 226 Hz. Literature has shown that tympanometry with a single probe tone has poor sensitivity and specificity in identifying middle ear pathologies (Kaf, 2011; Sebothoma & Khoza-Shangase, 2018). For example, Hunter et al. (2017) found that tympanometry with a single probe tone missed approximately 20% of CHL in children. These findings suggest that further research in children living with HIV must use measures that have high sensitivity and specificity in identifying middle ear pathologies. Wideband acoustic immittance (WAI) has emerged as the potential middle ear measure with higher sensitivity in identifying early signs of middle ear pathologies compared with the conventional tympanometry with single probe tone (Kaf, 2011; Sebothoma & Khoza-Shangase, 2018; Shahnaz et al., 2009; Terzi et al., 2015).

While otomicroscopy is considered gold standard in identifying middle ear pathologies in some countries (Sebothoma & Khoza-Shangase, 2018), the shortages of otolaryngologists in LMICs (Christopher et al., 2013), limit the practicality of this measure. Consequently, video otoscopy used asynchronously has also been shown to be equally sensitive in identifying middle ear pathologies compared with otomicroscopy. Martines et al. (2010) found a substantial agreement (*k* = 0.7) between the diagnoses made from video otoscopic images and those from onsite otomicroscopy. Sebothoma and Khoza-Shangase (2018) also demonstrated that video otoscopy can produce accurate diagnosis of the middle ear pathology than the conventional tympanometry with single probe tone. It is therefore crucial that future studies include some of these sensitive tests to provide a more accurate picture of the prevalence and nature of middle ear pathologies in children living with HIV.

In this review, HL with a conductive component was found to be the second most reported type of HL in children living with HIV (Christopher et al., 2013). The prevalence of this type of HL was reported to be 36%, with the severity reaching moderate levels (Christopher et al., 2013). Given the fact that CHL was the second most reported type of HL, Dawood et al. (2020) suggest that this type of HL may be associated with otitis media with effusion (OME). In studies reviewed, the prevalence of OME was found to be 6.8% (Martines et al., 2010). These findings are consistent with the study by Christopher et al. (2013), who found that HL with a conductive component in children is likely because of OME, which is one of the common types of middle ear pathologies in children (Vanneste & Page, 2019).

Human immunodeficiency virus-positive children were shown to have more signs and symptoms related to middle ear pathologies compared with children without HIV. These symptoms or signs included otalgia, tympanic membrane perforation, otorrohea and impacted wax (Hrapcak et al., 2016; Maro et al., 2016; Mwambete & Eulambius, 2018; Taipale et al., 2011; Torre et al., 2016). Of all these signs or symptoms, otorrohea, tympanic membrane perforation, history of ear infections, low immune system and WHO stage IV of HIV were associated with middle ear pathologies in children living with (Dawood et al., 2020; Hrapcak et al., 2016; Maro et al., 2016; Mwambete & Eulambius, 2018; Taipale et al., 2011; Torre et al., 2016). Studies on adults living with HIV also reported that middle ear pathologies result from opportunistic infections because of a low immune system resulting from a declined number of CD4 T cells (Obasineke et al., 2014; Van der Westhuizen et al., 2013). However, in one study (Torre et al., 2016), middle ear pathologies were only associated with flat tympanogram and otorrohea. The difference between previous studies and findings in this study by Torre et al. (2016) may be because of different sample sizes. Therefore, these conflicting results warrant further research in order to determine the contributing factors for middle ear pathologies.

Despite the paucity of evidence, this review suggests that middle ear pathologies exist in children living with HIV and may cause HL. Therefore, there is a need for further research to identify the significant relationships or differences that exist between studies. Conducting studies on middle ear pathologies will further enhance our knowledge regarding the middle ear function of children living with HIV and help to provide appropriate services for this population. Early identification of middle ear pathologies is significant for all children, especially those with a compromised immune system. Middle ear pathologies are known to have a negative impact on children’s language and quality of life (Torre et al., 2016). For example, recurrent otitis media (ROM) in children is associated with phonological delay (Torre et al., 2016) and because children with HIV are at a high risk of ROM, it is necessary to identify early signs of their middle ear pathologies so that timeous interventions can be implemented early. Failure to implement early identification and intervention for children living with HIV may contribute to delays in schooling.

For example, about 13% of children with HL and living with HIV never get a chance to attend school (Hrapcak et al., 2016). While some children living with HIV already experience difficulty attending school because of constant sickness (Hrapcak et al., 2016), very few of those who are able to attend school get to perform above average because of HL related to middle ear pathologies. Therefore, it is crucial that children living with HIV receive early identification and intervention for middle ear pathologies. This is even more crucial in LMICs where resources for assessing and managing middle ear pathologies are extremely limited (Fagan & Jacobs, 2009).

## Conclusion

The studies reviewed in this scoping review provided evidence that middle ear pathologies in children living with HIV exist and may be severe or chronic, causing HL to various degrees. However, because of the limited number of articles found during this scoping review, the findings must be interpreted with caution. While the studies reviewed provided some important information about middle ear pathologies in children living with HIV, all the studies used measures that have been shown to have poor sensitivity and specificity. Further research using sensitive measures of middle ear pathologies such as WAI and with large sample sizes must be conducted. Furthermore, studies must use longitudinal designs in order to determine the change in middle ear function and pathologies (Sebothoma & Khoza-Shangase, 2020).
